# Location and Electronic Nature of Phosphorus in the Si Nanocrystal − SiO_2_ System

**DOI:** 10.1038/srep09702

**Published:** 2015-05-22

**Authors:** Dirk König, Sebastian Gutsch, Hubert Gnaser, Michael Wahl, Michael Kopnarski, Jörg Göttlicher, Ralph Steininger, Margit Zacharias, Daniel Hiller

**Affiliations:** 1 Integrated Material Design Centre (IMDC), UNSW, Sydney, Australia; 2 School of Photovoltaic and Renewable Energy Engineering (SPREE), UNSW, Sydney, Australia; 3 Laboratory of Nanotechnology, Dept. of Microsystems Engineering (IMTEK), University of Freiburg, Germany; 4 Department of Physics and Research Center OPTIMAS, University of Kaiserslautern, Germany; 5 Institute for Surface and Thin Film Analysis (IFOS) Ltd., Kaiserslautern, Germany; 6 ANKA Synchrotron Radiation Facility, Karlsruhe Institute of Technology, Germany

## Abstract

Up to now, no consensus exists about the electronic nature of phosphorus (P) as donor for SiO_2_-embedded silicon nanocrystals (SiNCs). Here, we report on hybrid density functional theory (h-DFT) calculations of P in the SiNC/SiO_2_ system matching our experimental findings. Relevant P configurations within SiNCs, at SiNC surfaces, within the sub-oxide interface shell and in the SiO_2_ matrix were evaluated. Atom probe tomography (APT) and its statistical evaluation provide detailed spatial P distributions. For the first time, we obtain ionisation states of P atoms in the SiNC/SiO_2_ system at room temperature using X-ray absorption near edge structure (XANES) spectroscopy, eliminating structural artefacts due to sputtering as occurring in XPS. K energies of P in SiO_2_ and SiNC/SiO_2_ superlattices (SLs) were calibrated with non-degenerate P-doped Si wafers. 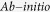 results confirm measured core level energies, connecting and explaining XANES spectra with h-DFT electronic structures. While P can diffuse into SiNCs and predominantly resides on interstitial sites, its ionization probability is extremely low, rendering P unsuitable for introducing electrons into SiNCs embedded in SiO_2_. Increased sample conductivity and photoluminescence (PL) quenching previously assigned to ionized P donors originate from deep defect levels due to P.

About 60 years ago, impurity doping of bulk Si was established to introduce majority charge carriers, creating p/n junctions as fundamental building blocks of Si-based electronic devices. The discovery of size-controlled solid-state growth of SiNCs from Si-rich SiO_2_ (SiO_*x*_)^1^ led to discussions about conventional dopants in SiNC/SiO_2_ systems. P is of particular interest due to high solubility and diffusivity in Si^2^. Detailed insight into the behaviour of P within the SiNC/SiO_2_ material system is crucial. It clarifies whether conventional SiNC doping is able to further advance miniaturization of Si-based electronic structures and electronic SiNC manipulation.

Many works have claimed doping of SiNCs with P donors^3^,^4^,^5^,^6^,^7^,^8^, but very few provided unambiguous evidence and detailed data on doping probabilities^9^,^10^ as gauge for working (active) dopants. Experimental evidence of successful P doping in SiNC/SiO_2_ samples like quantum dot solar cells^11^ or standard capacitance-voltage curves requiring a bulk semiconductor space charge region^12^ likely occur due to interconnected SiNC/amorphous Si networks^13^ where conventional doping does work to some extent. SiNCs separated by ultrathin SiO_2_ barriers are dominated by defect-assisted conduction^14^, though electric conductivities can be tremendously increased by massive P incorporation in the 0.5 to 8 atom-% range (0.25 to 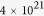 cm^−3^)^3^,^4^,^5^,^6^,^7^,^8^. Such high P concentrations enter the composition range of ternary compounds (SiO_*x*_P_*y*_) with different properties as compared to Si, SiO_*x*_ and SiO_2_. We note that Pearson and Bardeen^15^ observed the semiconductor to metal transition of bulk Si for donor (P) and acceptor (boron; B) concentrations around 0.25 atom-% (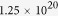 cm^−3^). With P concentrations in the 0.5 to 8 atom-% range, clustering with dopant inactivation, defect formation and massive out-diffusion occur already in bulk type Si layers for structure sizes of 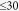 nm in ultra-large scale integration (ULSI)^16^,^17^. Local P density fluctuations in SiNCs prevent to provide exactly one active dopant per SiNC^18^. The vast majority of SiNCs are undoped and very few SiNCs have multiple dopants. Latter leads to significant random deterioration of their electronic properties by exchange coupling^19^. Massive P densities in SiNC systems lead to P localized in SiO_2_, in SiO_*x*_ surrounding SiNCs and P gettered by dangling bonds (DBs) at NC interfaces, all being critical for the electronic structure. So far, unpaired electrons bound to P were investigated by electron paramagnetic resonance (EPR) at very low temperatures^9^,^10^. Thermal broadening of EPR resonances prevented measurements at room temperature (*T* = 300 K). XANES is not restricted to low temperatures and yields information on the electronic state of *all* P at *T* = 300 K. Excited P K shell electrons in XANES have tremendously increased mean free paths as compared to X-ray photoelectron spectroscopy (XPS) due to their high kinetic energy 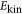
20. We boosted sampling depths further by using XANES in fluorescence yield mode, allowing for non-destructive probing depths three orders of magnitude above XPS values. Due to the low 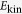 of P L-III shell electrons, XPS is extremely surface sensitive. Probing samples below their original surface by XPS requires sputtering off top material, introducing artefacts as function of chemical species like sputter yield and atom re-coordination and re-ordering.

We report on h-DFT calculations of P at central lattice and interstitial sites in completely OH-terminated SiNCs, of saturated P at the surface of such NCs, in SiO_0.9_ as sub-oxide shell around SiNCs and in SiO_2_, delivering insights into the specific electronic structure due to P. The spatial distribution of P atoms in SiNC/SiO_2_ systems is derived from APT data and their statistical processing to yield the P distribution profile from the SiO_2_ matrix to the interior of the SiNCs. We discuss P data from h-DFT and XANES together with P spatial statistics from APT and obtain a detailed picture of the electronic behaviour of prospective P donors depending on their positions and bond geometries in SiNC/SiO_2_ systems. The 1s core level energies from h-DFT are used to assign XANES signals to respective P configurations in h-DFT approximants.

## Results

### Hybrid DFT calculations

**Figure 1:**
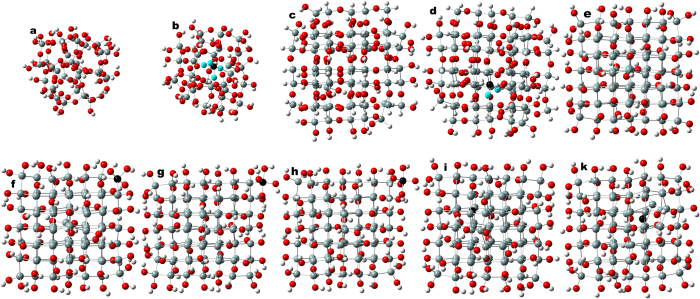
Optimized approximants calculated by h-DFT. Top row shows SiO_2_ reference – Si_29_O_40_(OH)_36_ (**a**), SiO_2_:P – Si_28_PO_40_(OH)_36_ (**b**), SiO_0_._9_ reference – Si_74_O_55_(OH)_35_H_29_ (**c**), SiO_0_._9_:P – Si_73_PO_55_(OH)_35_H_29_ (**d**) and fully OH-terminated 15 Å NC reference OH-SiNC – Si_84_(OH)_64_ (**e**). The bottom row shows OH-SiNC with corner >Si(OH)_2_ substituted by >P(OH)_3_ referred to as OH-SiNC>P(OH)_3_ – Si_83_P(OH)_3_(OH)_62_ (**f**), with OH group at corner Si atom substituted by P(OH)_4_ referred to as OH-SiNC-P(OH)_4_ – Si_84_P(OH)_4_(OH)_63_ (**g**), with OH group at corner Si atom substituted by OP(OH)_4_ referred to as OH-SiNC-O-P(OH)_4_ – Si_84_OP(OH)_4_(OH)_63_ (**h**), OH-SiNC with central Si atom substituted for tetravalent P referred to as OH-SiNC-P[Si] – Si_83_P(OH)_64_ (**i**), and with P on an interstitial site in the NC center referred to as OH-SiNC-P[is] – Si_84_(OH)_64_-is-P (**k**). Atom colors: Si is gray, P is black, O is red and H is white. 1-nn O atoms of P in SiO_2_ and SiO_0_._9_ approximants shown in cyan. Approximants shown along [001] lattice vector group except SiO_2_ and SiO_2_:P.

Figure 1 shows optimized approximants of a SiO_2_ reference (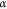-quartz), of SiO_2_ with P on a central Si site (SiO_2_:P), of a SiO_0.9_ reference and of P-doped SiO_0.9_ (SiO_0.9_:P). Fig. 1 further shows optimized approximants of a fully OH-terminated SiNC of 15 Å size as NC reference (OH-SiNC), and this NC with saturated (penta-valent) P substituting a corner Si atom (OH-SiNC 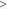 P(OH)_3_), an OH group on such corner Si atom (OH-SiNC-P(OH)_4_) and a H atom at the OH group substituted by P(OH)_4_ (OH-SiNC-O-P(OH)_4_). Fig. 1 also shows optimized approximants of fully OH-terminated 15 Å SiNCs with P on a central Si lattice site (OH-SiNC-P[Si]) and on a central interstitial cite (OH-SiNC-P[is]). Interstitial P coordinates relative to its 1-nn Si atoms were used from experiment^21^. Convergence of structural optimization of the approximant was accepted for residual forces on interstitial P and its 1-nn Si atom (and all other atoms) of 309 *μ*eV/Å (11.3 *μ*Ha/Å) which is ca. 1.3% of the convergence threshold of maximum residual forces, see to Methods section at end of article. The atomic displacement associated with this minute residual force was 0.0032 Å (0.32 pm) which is ca. 94% of the convergence threshold of residual displacements of 0.003403 pm – a rather large value for such residual force. This variance is an indication of a somewhat flat energy landscape. Thereby, it is rather difficult to calculate an exact diffusion path of interstitial P. This may explain why dopant atoms on Si lattice sites were considered in *ab-initio* thermodynamic diffusion simulations^22^,^23^,^24^,^25^, but dopant atoms on interstitial positions were not included. Further details on DFT calculations can be found in the Methods section at the end of the article.

### *Electronic Structure of P in SiO_2_.* 

**Figure 2:**
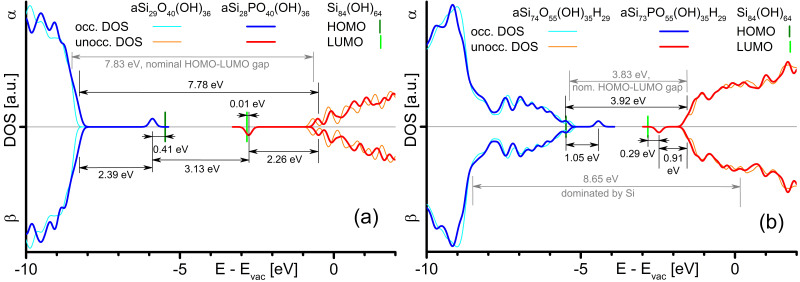
Electronic DOS of oxides containing P. Results for SiO_2_:P (**a**) and SiO_0.9_:P (**b**), shown with DOS of pure SiO_2_ (top) and pure SiO_0.9_ (bottom) approximants. Dark (bright) green lines show HOMO (LUMO) of OH-SiNC.

The SiO_2_ HOMO-LUMO gap is 7.83 eV which is 89% of the experimental value of ca. 8.8 eV^26^. We consider P on tetragonal Si sites in SiO_2_ and SiO. P is surrounded by SiO_2_ at least to its 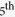 next neighbour (5-nn) atom. Oxidation enthalpies^27^ are 916 kJ/mol (9.49 eV/Si atom) for the chemical reaction Si + O_2_ → SiO_2_ and 1493 kJ/mol (7.74 eV/P atom) for the reaction 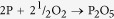, indicating that pentavalent P configurations (P(–O–)_5_) should not be favoured over tetravalent Si (Si(–O–)_4_), leaving P with a DB in analogy to P donors in bulk Si. The DB of P is strongly associated with 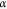-HOMO and 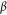-LUMO, describing one state with its two spin configurations 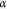 and 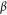 (Fig. 2a). We compare the energies of frontier MOs with HOMO and LUMO energies of the OH-SiNC reference (Fig. 2, green lines). The 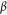-LUMO energy of SiO_2_:P is 0.01 eV above the LUMO of the OH-SiNC approximant while the 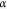-HOMO of SiO_2_:P is 0.41 eV below the HOMO of the OH-SiNC approximant. The barrier height for electron (hole) transport is given by the conduction (valence) band offset between Si and SiO_2_ of 3.2 eV (4.5 eV)^26^. These values show that P in SiO_2_ reduces the transport barrier for electrons (holes) by 97% (85%), causing an extreme increase in electron conductivity and a considerably increased hole conductivity. These defect levels are an important electronic aspect of P in SiO_2_: It causes a massive increase in SiO_2_ conductivity while *not working* as a donor. Several works build their evidence of SiNC doping on conductivities increasing with P concentrations of 0.5 to 8 atom-%^3^,^4^,^6^,^7^,^8^. From the SiO_2_:P approximant we get an atomic ratio of P/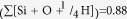 atom-% P, whereby we consider H terminating outermost O bonds as 1/4 Si.

### Electronic Structure of P in SiO

Approximants for SiO_0.9_ and SiO_0.9_:P are based on 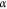-quartz. Every second O bridge Si–O–Si is substituted by a bond Si–Si. As with SiO_2_:P, we have a DB on P occupied with one electron in the SiO_0.9_:P approximant at a central Si lattice site, again resulting in two different spin orientations per MO (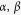). Frontier MOs are similar to SiO_2_:P, describing the DB of P with one electron occupying the 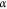-HOMO. The 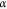-HOMO – 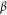-LUMO gap of 1.96 eV is 0.76 eV below *E*_*gap*_=2.72 eV of the OH-SiNC reference. The HOMO in SiO_0.9_:P is located 1.05 eV above the HOMO of the OH-SiNC reference. Hence, P presents a deep recombination center in SiO_*x*_ shells (Fig. 2b) which cover SiNCs with a thickness of 1 to 1.5 mono layers (MLs)^28^. This finding is supported by PL quenching reported for high P concentrations mentioned above^3^,^29^.

The LUMO of SiO_0.9_:P facilitates electron transport by diminishing the electron barrier. As for the SiO_2_:P approximant, electron (hole) barriers are decreased down to 32% (removed completely). For SiO_0.9_ and SiO_0.9_:P approximants, a helical arrangement of Si atoms along the 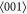 vector (Fig. 1c,d) dominates MOs from *E − E*_*vac*_ = 0.2 to -8.5 eV. The inner bonds of these Si backbones can resist electron transfer to O to some extent, diminishing the splitting of their bonding and anti-bonding MOs. Experiments yield 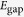
(SiO) ≈ 2.48 eV^30^, our calculations overestimate this value by 54%. This may be due to the very balanced local stoichiometry of the SiO_0.9_ reference and SiO_0.9_:P approximants as well as their high space group symmetry which allows for mentioned Si helices. Local Si segregation suggests that SiO is not uniform^13^ which can lower the band gap. The P concentration can be calculated as for the SiO_2_:P approximant, yielding 0.56 atom-% for SiO_0.9_:P.

### Electronic Structure: Saturated P at SiNC interfaces

Tetravalent P atoms substantially gain binding energy when gettering their DBs at NC interfaces and maximize binding energies of Si atoms providing DBs. It is thus energetically unfavourable for P at the NC interface to have a DB. This finding is supported by a maximum P density at SiNC interfaces derived from APT below.

**Figure 3:**
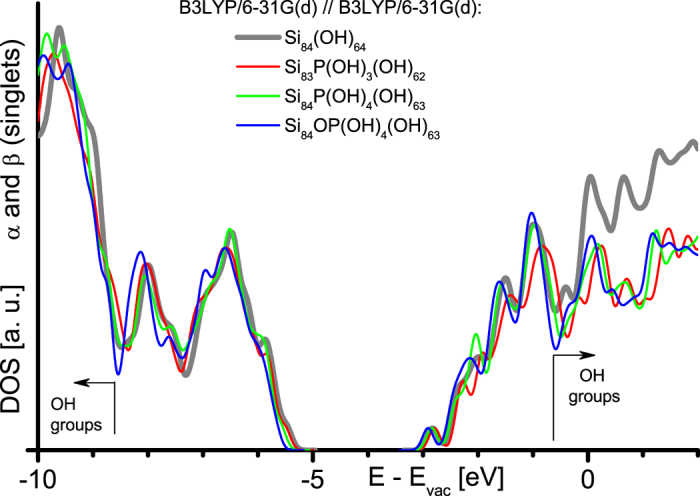
Electronic DOS of SiNCs with P at interface. Data of OH-SiNC reference (Si_84_(OH)_64_) and its versions with bond-saturated P at NC interface, see Figure 1 for details.

We show the DOS of the OH-SiNC reference approximant along with the DOS of all three approximants containing bond-saturated P at the interface (Fig. 3). Fully gettered P at NC interfaces does not introduce defect levels within the HOMO-LUMO gap of the SiNC. The DOS of OH groups has an energy gap of 8.0 eV, corresponding to 91% of the experimental band gap of SiO_2_^26^. The DOS of the SiNC approximants expose a small shift of HOMO and LUMO to higher binding energies, correlating with an increasing number of O atoms^31^,^39^.

### Electronic Structure of P within SiNCs

**Figure 4:**
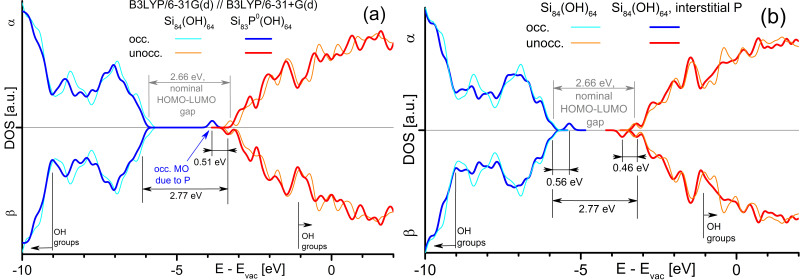
Electronic DOS of OH-SiNC approximant with P residing inside SiNC. P located on a central Si substitutional site (**a**) and on a central interstitial site (**b**), shown with DOS of reference OH-SiNC approximant.

We consider P on a central Si lattice site OH-SiNC-P[Si] and on a central interstitial site OH-SiNC-P[is]. P on a Si lattice site generates a HOMO 0.51 eV below the LUMO energy (Fig. 4a). While this HOMO presumably becomes a donor state for vanishing quantum confinement, its ionization energy *E*_ion_=0.51 eV is too big to ionize SiNCs with a reasonable probability at *T *= 300 K; 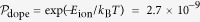. Even for SiNCs at the upper size limit of quantum confinement, 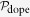 will be too small for providing electrons to SiNCs; experimental values^10^ for 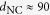 Å are 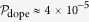. Interstitial P introduces two gap states, a HOMO 0.57 eV above the HOMO of the 1.5 nm SiNC and a LUMO 0.46 eV below the LUMO of the SiNC (Fig. 4b). Both states due to P cannot donate electrons but provide efficient carrier recombination with a transition energy of 1.72 eV. As this transition is optically active at a wavelength of ca. 720 nm, it must be considered for PL spectra of P-doped SiNC/SiO_2_ species. Both cases of P in OH-SiNC introduce recombination levels into SiNCs.

### Atom Probe Tomography

**Figure 5:**
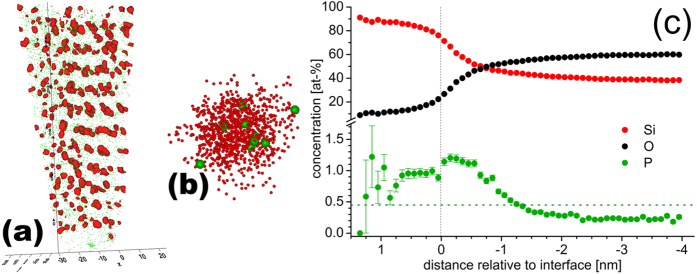
P-doped SiNC SL in SiO_2_ scanned by APT. Composition of SL, volumes with ≥70 atom-% Si are covered by red iso-surfaces, individual P atoms are shown in green (**a**). P atoms within 3 nm SiNC (**b**). Proxigram derived from SiNCs in left graph, showing radial concentration distribution of Si, O and P, latter with error bars for standard deviation (**c**). Zero of distance scale defined by interface located at SiO_0.3_ (85 mol-% Si and 15 mol-% SiO_2_, ignoring P content). Concentrations scanned along normal vector of interface into SiNCs, stopping at center of smallest SiNCs (size ca. 2.7 nm) to avoid signal back-folding. Horizontal dashed line shows average P concentration.

We show the APT scan of a SiNC SL in SiO_2_ where SiNCs are enclosed by iso-surfaces with atomic concentrations of Si 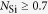, i.e. 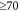 atom-% Si (Fig. 5a). With the molar ratio of 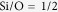 in SiO_2_, we derive the molar SiO_2_ partition 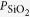 of SiNCs via 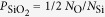 Ignoring the P partition of ca. 1 atom-%, we get 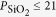 mol-% SiO_2_ and 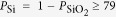 mol-% Si for volumes enclosed by iso-surfaces. We note that the real 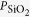 value is lower due to APT projection artefacts. Detailed statistical analyses of APT data^32^ revealed that about 15% of the P atoms are found within SiNCs, whereas about 30% are trapped at the interface and about 55% reside in the surrounding SiO_2_ matrix. This relatively low P concentration in SiNCs can be explained by self-purification^22^,^23^,^24^,^25^, by solubilities of P in Si and SiO_2_ and by the high relative SiO_2_ volume of 85% in our samples. Zooming into the APT scan shows P atoms within SiNCs (Fig. 5b). A notable P concentration within SiNCs appears to disprove self-purification. However, interstitial P^21^ should have a much higher probability to exist in SiNCs as compared to P built into SiNC lattice sites. It does not require bond breakage and can exploit the fast diffusivity and high saturation density of P. An inclusion of such P configurations into *ab-initio* thermodynamic diffusion simulations would complement existing self-purification models which only consider foreign atoms at SiNC lattice sites. Tomogram data from APT used for a cluster analysis^32^ comprised numerous SiNCs in SiO_2_:P. The resulting proxigram shows the radial concentration of Si, O and P (Fig. 5c). We found a strong accumulation of P atoms in the SiNC/SiO_2_ interface shell with SiO_*x*≈1_ and also an increased P concentration within SiNCs.

### XANES spectroscopy

**Figure 6:**
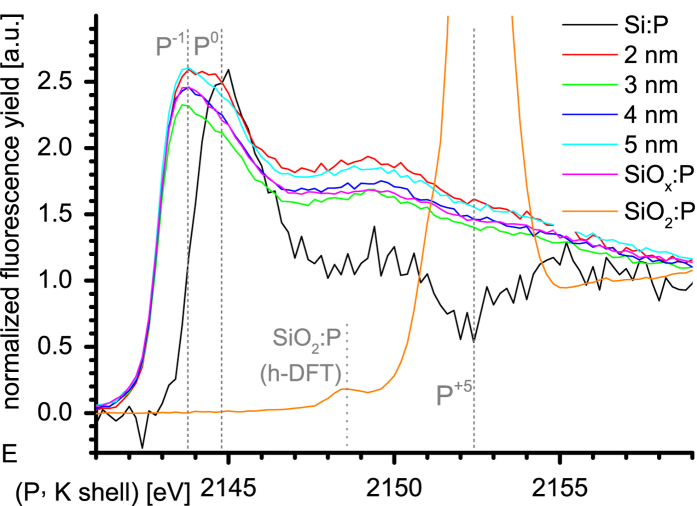
XANES spectra of SiNC/SiO_2_ samples. Normalized K shell spectra of P in SiNC/SiO_2_ SLs (2 nm, 3 nm, 4 nm, 5 nm), annealed bulk SiO_*x*_ sample SiO_*x*_:P and P doped SiO_2_ sample SiO_2_:P shown together with doped Si wafer Si:P. Dashed gray lines show P oxidation stages.

**Figure 7:**
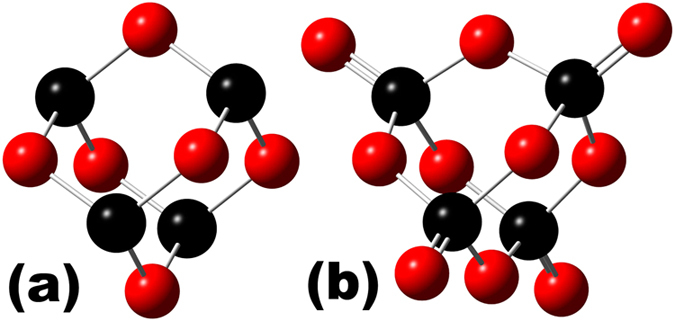
P_2_O_3_ and P_2_O_5_ approximants for XANES calibration. Approximants of P_4_O_6_ (a) and P_4_O_10_ (**b**) cages constituting P_2_O_3_ (P^+3^) and P_2_O_5_ (P^+5^), respectively^27^.

We measure P K spectra to determine the P oxidation stage by its K shell electron binding energy (Fig. 6), using a non-degenerate P-doped Si wafer (donor density = 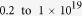 cm^−3^ or 0.004 to 0.02 atom-%) for calibration. We assign XANES results to P environments using 1s core levels calculated by h-DFT with all-electron MO-BSs. P 1s core level energies from h-DFT correspond to 97.864% of P K XANES energies, see table 1. We calibrated h-DFT values by a factor of 1.02183 as supported by h-DFT P 1s core level energies of P_2_O_5_ (P^+5^) and P_2_O_3_ (P^+3^) approximants calculated with the same h-DFT route (Fig. 7).

## Discussion

### Origin of PL quenching of SiNCs containing P

Diffusion of P through Si proceeds at high rates during SiNC segregation anneal with 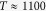 °C. Experimental data shown above and DFT calculations^22^,^23^,^24^,^25^ indicate that P appears to be within SiNCs on interstitial sites with a probability of nearly 100%. Auger recombination was assumed to cause PL quenching in SiNC/SiO_2_ material systems with high P concentrations^3^. Our findings do not support this assumption. With extremely low P ionization probabilities, the difference in free carrier densities of doped and intrinsic SiNCs is virtually nil. The Auger recombination 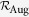 rate is^33^,^34^ 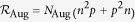, where 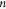 (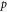) are the density of free electrons (holes) and 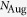 is the Auger scattering coefficient (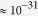 cm^6^/s for bulk Si^34^). Under high injection conditions (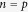), Auger recombination is 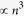 which explains its strong increase at high free carrier densities^35^,^36^. P located within SiNCs or within SiO_*x*_ shells around SiNCs are deep defect centers which appear to provide the most efficient and fastest path for non-radiative carrier recombination. This process explains PL quenching already at reasonably high P densities^29^ still below values reported elsewhere^3^,^4^. Co-doping with B was shown in experiment to 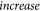 PL intensities while transition energies decreased below those of undoped SiNCs^3^. Localized states of B and P at or within SiNCs provide strong radiative transitions as donor electrons directly relax into acceptor states. PL energies decreasing with co-doping 37 are a clear indication of this mechanism.

### P ionization in SiNC/SiO_2_ samples

P in bulk Si has four bonds to its 1-nn Si atoms, acquiring 0.09 electrons (2.2% bond ionicity). The P charge is 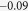 for neutral donors and 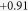 for ionized donors. P donors in bulk Si have 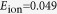 eV^38^, yielding a doping (ionization) probability at *T* = 300K of 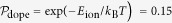. The average charge of all P atoms in bulk Si is then 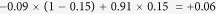, corresponding to oxidation stage zero (P^0^). This value refers to the XANES peak at 2144.8 eV of the P doped Si wafer reference (Si:P), see Fig. 6. All P-doped SiNC/SiO_2_ samples (2 to 5 nm SiNC/SiO_2_ SLs, bulk) show peaks at 2143.7 eV. The 1.1 eV shift to lower binding energies shows that P in SiNC/SiO_2_ is much less positively ionized, corresponding to P^−1^. This result is corroborated by the Mulliken charges of P obtained from h-DFT and the analytical value of P in bulk Si (table 1). A hint of a signal shoulder might exist for all SiNC samples at the XANES peak for P^0^ at 2144.8 eV. An indication of a signal occurs for the smallest SiNC size of 2 nm, suggesting a slightly increased doping probability for ultrasmall SiNCs also observed by EPR^10^, though the ultrasmall SiNC size notably increases the signal background for XANES and presumably EPR. Our results show that P does not provide electrons to SiNCs embedded in SiO_2_ with reasonable probabilities.

## Conclusion

We carried out DFT calculations for the SiNC/SiO_2_ system to monitor the electronic nature of P. On a lattice site within OH-terminated SiNCs, P introduces a deep donor level with E_*ion*_ = 0.51 eV; ionisation for small SiNCs is virtually nil, but is likely to increase for SiNCs with diminishing quantum confinement. However, formation energies of P on Si lattice sites^22^,^23^,^24^ suggest that P in SiNCs occurs almost exclusively on interstitial sites which is indirectly corroborated by experiments showing an extremely small density of P atoms with unpaired electrons even for 10 nm SiNCs^9^,^10^. On a central interstitial site within SiNCs, P cannot donate an electron (E_*ion*_ >2 eV), but forms two deep defect levels with a recombination transition at 1.72 eV. At SiNC interfaces, fully saturated P have no impact on frontier molecular orbitals, leaving HOMO and LUMO energies virtually unchanged. In SiO shells around SiNCs, P is again unable to donate an electron, but induces a deep defect level which triggers massive recombination. This defect causes PL quenching – as opposed to Auger recombination – and increases SiO shell conductivities which were both interpreted as evidence for successful SiNC doping in the literature^3^,^8^. Although P atoms in SiO_2_ are deep defects which cannot donate electrons, they tremendously improve inter-NC conductivities in particular for electrons by diminishing electron (hole) barriers by 97% (85%) of the conduction (valence) band offset between bulk phases of Si and SiO_2_. Massively increased conductivities were assumed to prove successful SiNC doping^6^,^8^. APT analyses revealed an enrichment of P at SiNC interfaces, which appears to be due to DB saturation and support h-DFT analyses of fully O-saturated P at SiNC interfaces. SiNCs were found to contain significant amounts of P. While this appears to contradict self-purification theory, interstitial P with considerably more favourable thermodynamics and its high diffusivity and saturation density has not been considered in self-purification modeling. Core level (K shell) electron energies of P in SiO_2_ and SiNC/SiO_2_ samples were measured by XANES at room temperature. In contrast to bulk Si, P atoms in SiNC/SiO_2_ samples could not donate electrons into 2 to 5 nm size NCs in SLs or in annealed bulk SiO_*x*_ films with reasonable probabilities, confirming our h-DFT results. We conclude that conventional doping of SiNCs with P does not provide majority charge carriers to SiNCs embedded in SiO_2_. Alternative approaches for majority carrier introduction into embedded SiNCs and ultrasmall Si nanovolumes such as embedding material effects^39^ have to be explored to advance SiNC-based nanoelectroncis and ULSI.

## Methods

### Sample Preparation

Size-controlled SiNCs in SiO_2_ were fabricated by deposition of P-doped Si-rich oxide (SiO_0.93_)/ intrinsic SiO_2_ SLs by plasma enhanced chemical vapor deposition and subsequent annealing (1150 °C, 1 h). During deposition, P was incorporated by adding 1% PH_3_ to Ar, resulting P concentrations were ca. 1 atom-% as found by secondary ion mass spectroscopy (SIMS)^29^,^32^. All samples were fabricated on low B-doped Si wafers (20 Ωcm) with 30 nm SiO_2_ layers to prevent P diffusion into Si substrates during anneal. For APT, P doped SLs with 30 bilayers and 5 nm nominal NC size were fabricated. Samples with 50 bilayers and nominal NC sizes from 2 to 5 nm in steps of 1 nm were chosen for XANES. In addition, 300 nm thick P-doped SiO_2_ and SiO_0.93_ samples were fabricated as references.

### Hybrid Density Functional Theory (h-DFT) Calculations

Approximants were calculated with non-periodic boundary conditions and underwent geometrical optimization with the B3LYP h-DF^40^,^41^ and the 6-31G(d) all-electron molecular-orbital basis set (MO-BS)^42^,^43^,^44^ using the GAUSSIAN 03 and GAUSSIAN 09 suites^45^,^46^. RMS and peak force convergence limits were 15.4 meV/Å (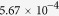 Ha/Å) and 23.1 meV/Å (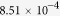 Ha/Å), respectively. Electronic structures were computed with the same route; B3LYP/6-31G(d) // 
B3LYP/6-31G(d). Additional information is available on accuracy tests and tests of functional group termination as approximation of the dielectric^31^,^39^,^47^. During all calculations, no MO symmetry constraints were applied and tight convergence criteria were set for the self-consistent field routine.

Alternative approaches to the B3LYP h-DF with similar accuracy are the Heyd-Scuseria-Ernzerhof h-DF (HSE06)^48^ and the Becke-Johnson exchange potential^49^,^50^, latter used within the Perdew-Burke-Ernzerhof (PBE) DF^51^ generalized gradient approximation (GGA) scheme.

### Characterisation

We examined the position of P within the SiNC/SiO_2_ system by APT using a Cameca LEAP 4000X HR instrument with a reflectron-type time-of-flight mass spectrometer and a pulsed UV laser (355 nm, 10 ps pulse length, 70 pJ pulse energy, 100 kHz repetition rate). During the analyses (chamber pressure 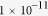 mbar), specimens were cooled to temperatures of around 76 K. The mass resolution of the system was 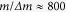, around 36% of all atoms are detected. Specimen tips have been prepared by the cut-and-lift-out technique using an ALTURA 875 dual-beam Focused Ion Beam instrument^32^.

The P K-edge absorption in XANES was measured at the SUL-X beamline at the Angströmquelle Karlsruhe (ANKA). Monochromatic X-rays were obtained using a Si(111) double crystal monochromator with an energy resolution of about 0.2 eV at 2150 eV with fixed exit. Scans were carried out using a shallow incident angle to maximize the SL or thin layer volume of samples for excitation. Absorption was measured by monitoring the P K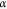 fluorescence emission using a seven element Si(Li) fluorescence detector (SGX Sensortech). The signal is normalized to the incident photon flux measured simultaneously by a custom made ionization chamber (ADC, US) filled with N_2_ at a pressure of 50 mbar. Energies were calibrated to 2152 eV at the white line maximum of the P K-edge XANES spectrum of NaH_2_PO_2_ ⋅ 2 H_2_O. The energy step size across the XANES region was 0.2 eV. XANES peaks of our samples show a full width half maximum of ca. 2 eV. P K XANES spectra have been pre- and post-edge background corrected and normalized to the edge jump with the ATHENA program of the IFEFIT package^52^.

## Additional Information

**How to cite this article**: König, D. *et al.* Location and Electronic Nature of Phosphorus in the Si Nanocrystal – SiO_2_ System. *Sci. Rep.* **5,** 9702; doi: 10.1038/srep09702 (2015).

## Figures and Tables

**Table 1:** Core level energies of P (1s from h-DFT, K shell from XANES). Bold numbers present XANES values, underlined numbers indicate approximants with same or similar configuration to samples indicated by arrow.

*E* (eV)	*q*(*e*^a^)	samples/	1-nn O of P;	remarks
**(XAS, DFT)**		**approximant**	**valence state**^b^	
2152.4		SiO_2_:P (major)	5; V	also assigned to O=P(–O–)_3_[53]
2150.8	+1.98	P_4_O_10_ cage (2 P_2_O_5_)	4; V	
2149.4	+1.42	OH-SiNC-O-P(OH)_4_	5; V	charge transfer to –**O**–P(OH)_4_
2148.6Ø	+1.29	SiO_2_:P	4; IV	P in center with DB
2148.6		SiO_2_:P (minor)	4; IV	
2148.3	+1.17	OH-SiNC-P(OH)_4_	4; V	P directly on NC, four OH on P
2147.5	+1.02	P_4_O_6_ cage (2 P_2_O_3_)	3; III	fully occupied 3sØ≠ AO on P
2146.8	+0.94	OH-SiNC>P(OH)_3_	3; V	P Si NC corner, >Si(OH)2→>P(OH)_3_
2146.0	+0.51	SiO_0.9_:P	2; IV	P in center with DB
2144.8	+0.06	P in bulk Si	0; IV	DB on P, 15% donors (P^+0.06^)
2144.5Ø	−0.28	OH-SiNC-P[Si]	0; IV	P with DB on Si site in NC center
2143.7		P in SiNCs	0; ??	all SiNC sizes, SL and bulk SiO_*x*_ samples
2142.9≠	−0.35	OH-SiNC-P[is]	0; 0	interstitial P(3s≠Ø, 3p_≠0≠+1≠−1_), NC center

Further shown are P atomic charges, P configuration (DFT approximant, XANES sample), number of P 1
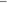
nn O atoms, P valence state and remarks.

^a^Values are Mulliken charges for DFT approximants and derived analytically for P in Si wafer (see text).

^b^P valence state is zero (0), tri (III), tetra (IV), penta (V) or unknown (??)
